# Intact habit learning in work addiction: Evidence from a probabilistic sequence learning task

**DOI:** 10.1016/j.abrep.2025.100589

**Published:** 2025-02-10

**Authors:** Zsuzsanna Viktória Pesthy, Krisztina Berta, Teodóra Vékony, Dezső Németh, Bernadette Kun

**Affiliations:** a Doctoral School of Psychology, ELTE Eötvös Loránd University, Budapest, Hungary; b Institute of Psychology, ELTE Eötvös Loránd University, Budapest, Hungary; c Department of Education and Psychology, Faculty of Social Sciences, University of Atlántico Medio, Las Palmas de Gran Canaria, Spain; d Centre de Recherche en Neurosciences de Lyon, INSERM, CNRS, Université Claude Bernard Lyon 1, CRNL U1028 UMR5292, Bron, France; e BML-NAP Research Group, Institute of Psychology, Eötvös Loránd University & Institute of Cognitive Neuroscience and Psychology, Research Centre for Natural Sciences, Budapest, Hungary

## Abstract

Work addiction (WA) is characterized by excessive and compulsive working patterns that detrimentally affect the individual’s health and functioning. While prior studies have indicated an overreliance on habit learning in various addictions, this study is the first to examine its role in WA. 104 adults were categorized into low-risk and high-risk groups for WA based on their scores on the Work Addiction Risk Test. We used a probabilistic sequence learning task designed to assess habit learning through the implicit acquisition of structured patterns characterized by alternating sequences. No significant differences were observed between the groups, both in terms of accuracy and reaction time. These findings suggest that individuals with WA exhibit intact habit learning, indicating that the addictive nature of work behavior may not solely stem from habitual processes. This highlights the unique features of WA compared to other addictions, potentially contributing to the relatively better overall functioning observed in affected individuals.

## Highlights

• This is the first study investigating habit learning in work addiction.
• Our findings indicate that habit learning remains intact in work addiction.
• Increased compulsiveness in work addiction may not be linked to habitual processes.
• Work addiction shows distinctive habitual processes compared to other addictions.

## 1 Introduction

Individuals with behavioral addictions often persist in their addictive behaviors despite being aware of the long-term negative consequences and having made several unsuccessful attempts to quit (American Psychiatric Association, 2013, Furlong and Corbit, 2018). A key factor behind this compulsive pattern may be habit learning, a type of associative learning where repeated practice automates actions (Ostlund & Balleine, 2008). While everyday habits are generally adaptive, they can become rigid and inflexible in conditions like addiction and obsessive–compulsive disorder (OCD) (Furlong and Corbit, 2018, Gillan et al., 2011, McKim et al., 2016). This rigidity might extend to work addiction as well, a condition that has garnered increasing attention and shows considerable overlap with these disorders (Andreassen et al., 2016, Griffiths, 2005, Griffiths and Karanika-Murray, 2012). This raises the possibility that similar patterns of habit learning could play a role, which is a central focus of this research.

Work addiction, first conceptualized by Oates (1971) based on his own experiences, is defined as a compulsive drive to work excessively despite adverse consequences. Since then, various models have been proposed to examine its characteristics, each highlighting different aspects. However, the conceptualization and criteria for work addiction remain subjects of ongoing debate (e.g. Griffiths et al., 2018, Loscalzo and Giannini, 2017), with no consensus on its definition in the DSM-5 or the ICD-11 (American Psychiatric Association, 2013, World Health Organization, 2018). Many researchers, like Oates, underscore its obsessive–compulsive nature, noting substantial similarities with OCD (Ng et al., 2007, Robinson, 1989, Sussman, 2012). Expanding on these ideas, Loscalzo and Giannini (2017) proposed a dual-pathway framework combining addiction-related and obsessive–compulsive symptoms, with low work engagement as a key feature. They also suggested that work addiction might be conceptualized as a form of personality disorder (Loscalzo & Giannini, 2020).

However, an increasing body of research highlights the characteristics of work addiction as a behavioral addiction (Andreassen et al., 2012, Atroszko et al., 2019, Aziz et al., 2013, Clark et al., 2020). One prominent framework supporting this perspective is the component model, which identifies six core features of addictive disorders, applicable to work addiction as well (Griffiths, 2005, Griffiths and Karanika-Murray, 2012). These components include the centrality of work in the individual’s life (salience), using work as a coping strategy (mood modification), needing increasing amounts of work to achieve the desired effects (tolerance), experiencing unpleasant symptoms when unable to work (withdrawal symptoms), facing conflicts in various life areas due to excessive work (conflict), and the recurring patterns of excessive work despite negative consequences (relapse) (Griffiths & Karanika-Murray, 2012). In this study, we interpret work addiction within this theoretical framework as a form of behavioral addiction.

Work addiction affects an estimated 7–10 % of individuals in Europe and nearly 40 % in South Korea (Andreassen et al., 2014, Kang, 2020; Kun, Magi et al., 2020). Given its high prevalence, it is crucial to emphasize the significant consequences of work addiction, which adversely impact various aspects of life, including social, mental, and physical well-being (Chang et al., 2022, Griffiths et al., 2018). A recent *meta*-analysis (Kenyhercz et al., 2024) found that work addiction is strongly associated with work-life imbalance and diminished social functioning, manifesting as deteriorating relationships and increased work-family conflicts. Furthermore, work addiction is linked to serious health risks, including depression, burnout, heightened anxiety or increased substance use, and various somatic symptoms, such as cardiovascular issues or back pain (Atroszko et al., 2020, Kun et al., 2023, Matsudaira et al., 2013, Salanova et al., 2016, Serrano-Fernández et al., 2021). Research has consistently shown that work addiction is associated with increased impulsiveness, compulsiveness, negative affectivity, and lower self-esteem (Berta et al., 2023, Clark et al., 2016, Demetrovics et al., 2022; (Kun, Takacs et al., 2020). These characteristics suggest that work addiction shares notable similarities with other behavioral addictions, both in terms of personality traits, behavioral patterns, and consequences.

Although the cognitive aspects of work addiction remain largely underexplored, research findings linking work addiction to higher levels of impulsiveness, compulsiveness, rumination suggest a potential role for altered cognitive functioning (Berta et al., 2023, Clark et al., 2016, Demetrovics et al., 2022; (Kun, Takacs et al., 2020). To date, only one study has examined the neuropsychological underpinnings of work addiction (Berta et al., 2023). The findings indicate that certain goal-directed functions, such as inhibitory control and more complex working memory processes, are impaired in individuals with work addiction, whereas cognitive flexibility and simpler working memory tasks appear to remain intact. Building on these findings, the present study aims to explore the role of habit learning in work addiction, with the goal of enhancing our understanding of the cognitive processes underlying this behavioral addiction.

Habit learning is a highly adaptive process that underlies the development of automatic behaviors, such as driving a car, playing an instrument, or learning a language (Christiansen et al., 2012, Conway, 2020, Gillan et al., 2015). What differentiates compulsive and addictive behaviors from everyday habits is a stronger stimulus–response relationship and the dominance of habitual actions over goal-directed ones (Everitt and Robbins, 2016, Furlong and Corbit, 2018, McKim and Boettiger, 2015, Patrono et al., 2017). In conditions like addiction and OCD, which are characterized by compulsiveness, behaviors often become rigid and repetitive. This rigidity may result from excessive habit learning, frequently associated with deficits in reward processing (Figee et al., 2016, Gillan et al., 2016). Overactivation of the habitual system reinforces actions that have been repeatedly rewarded, facilitating the development of automatic behaviors that support efficient responses to routine tasks (Gillan et al., 2015). However, this same overactivation can lead to maladaptive and repetitive behaviors in situations requiring flexibility, a hallmark of compulsivity-related disorders (Doñamayor et al., 2022, Gillan et al., 2015, Patrono et al., 2017).

Several studies examined the habitual system in substance use disorders (Doñamayor et al., 2022, Ersche et al., 2016, Furlong and Corbit, 2018, Hogarth, 2018, Lim et al., 2019, McKim et al., 2016), yet research exploring the connection between behavioral addictions and the habitual system remains limited. However, existing studies suggest that habit learning may also play a significant role in behavioral addictions, such as internet addiction and gambling disorders (Wyckmans et al., 2019; B. Zhou et al., 2018; W. Zhou et al., 2021). These studies suggest that the predominance of habit learning may not only be a feature of substance use disorders but may extend to addictive disorders in general, even though it has been explored in only a few behavioral addictions. Given the shared behavioral and personality traits between work addiction and other behavioral addictions (Clark et al., 2016, Griffiths and Karanika-Murray, 2012; (Kun, Takacs et al., 2020), along with previously reported alterations in goal-directed processes in work addiction (Berta et al., 2023), suggest that, akin to other behavioral addictions, habitual processes may also play a dominant role in work addiction.

The aim of our study was to examine habit learning in work addiction, by comparing individuals with or without work addiction. While outcome devaluation tests are the most common method for assessing habit learning in addictions— how effectively individuals suppress a behavior after a stimulus is devalued (Doñamayor et al., 2022, Furlong and Corbit, 2018) sequential learning tasks provide a novel and effective approach examining habitual processes (Brezóczki et al., 2023, Doñamayor et al., 2022, Horváth et al., 2022). In this study, we utilized the Alternating Serial Reaction Time task (Howard & Howard, 1997), a probabilistic sequence learning paradigm, to explore these processes in the context of work addiction. By measuring the acquisition and automatization of recurring patterns in the environment (Armstrong et al., 2017), this methodology effectively models the process of habit formation, which is a fundamental mechanism underlying addiction development (Doñamayor et al., 2022, Horváth et al., 2022). We hypothesized that habit learning would be enhanced in participants with high risk for work addiction. This hypothesis is grounded in two key observations: (i) work addiction shares similar characteristics with addictive and OCDs (Andreassen et al., 2016, Griffiths and Karanika-Murray, 2012), (ii) and work addiction demonstrates the persistence of excessive work over the long term despite numerous negative physical, mental, and social consequences (Hakanen and Peeters, 2015, Salanova et al., 2016, Sussman, 2012).

## 2 Methods

### 2.1 Participants

A total of 108 Hungarian individuals were recruited for this study. Participation in the study required meeting specific criteria, including active employment, a minimum age of 18 years, and the absence of comorbid addictive disorders. Participants were excluded if they reported any comorbid addictive disorder (*n* = 4). In total, we analyzed 104 participants' data (*M*_age_ = 40.81 years; *SD* = 9.5, 64 females, 40 males). Based on their scores on the Work Addiction Risk Test (Robinson, 1989), participants were classified in the high risk and low-risk work addiction groups, using a standardized cutoff score of 67, which is widely employed in research (Robinson, 1999). Forty participants were categorized into the high risk for work addiction group (HWA, *M*_age_ = 38.15 years, *SD* = 8.24; 29 females, 11 males) while sixty-four were classified into the low risk group (LWA, *M*_age_ = 42.47 years, *SD* = 9.91; 35 females, 29 males). For the WART scores, the HWA group had a mean score of 74.93 (*SD* = 6.86) with a range of 25 (min. = 67, max. = 92), while the LWA group had a mean score of 56.31 (*SD* = 6.25) with a range of 25 (min. = 41, max. = 66).

In our final sample, 74.04 % of the participants resided in the capital city (*n* = 77), 19.23 % lived in another city or town (*n* = 20), 5.77 % were situated in a village or hamlet (*n* = 6), and 0.01 % a county seat city (*n* = 1). Regarding educational background, the majority of participants (85.58 %, *n* = 89) held a college or university diploma, 5.78 % had a doctoral degree (*n* = 6), 7.69 % completed their education with a high school diploma (*n* = 8), and one participant had completed vocational training without a high school diploma (0.01 %). During data collection, we also asked about the respondents' occupations. Two individuals did not respond to this question. Of the 100 respondents, 94 % (*n* = 94) were white-collar workers, 5 % (*n* = 5) were blue-collar workers, and 1 % (*n* = 1) was a working student who did not specify their occupation alongside their studies. In terms of occupational fields, 26.6 % worked in business, finance, and management; 11.9 % in arts, media, and design; 10.9 % in engineering and technical fields; 10.9 % in technology and IT; 9.9 % in healthcare and social services; 7.9 % in education; 7.9 % in law and public administration; 6.9 % in public services and other fields; 5 % in science and research; and 1 % was a student who did not provide further details about their employment.

### 2.2 Measures

#### 2.2.1 Alternating Serial Reaction Time task

To assess habit learning, we used the Alternating Serial Reaction Time task (ASRT; Howard & Howard, 1997), a probabilistic sequence learning task, which was implemented through a computerized version developed with the JavaScript jsPsych library (de Leeuw, 2015, Vékony, 2021). This task is measuring the learning and automation of environmental regularities, an important aspect of habit formation (Horváth et al., 2022).

Participants were unaware of a hidden pattern guiding the sequence of stimulus presentations: every first element followed a pattern, while the second appeared randomly (e.g., 3r2r1r4r, with numbers indicating patterned elements and “r” symbolizing the random elements that can appear in any of the four positions). Due to this alternating structure, some runs of three consecutive elements were more predictable (high-probability triplets) than other runs of three elements (low-probability triplets) (see further in Supplementary S1).

**Fig. 1:**
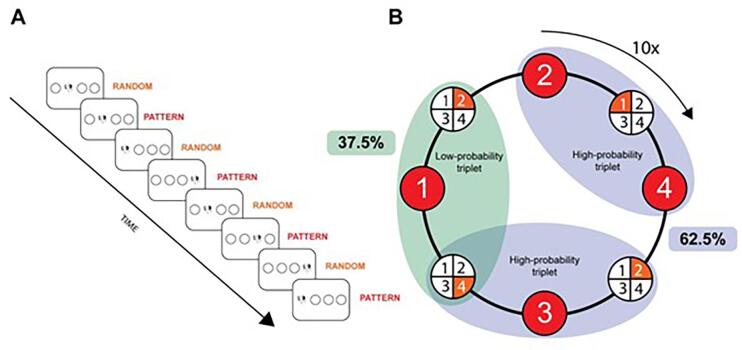
Structure of the ASRT task. *Note*. In this task, the random and pattern elements are following each other. From this alternating structure, we can get high-probability triplets (where the last element might be a part of the established pattern or could alternatively be selected from the random elements; 62.5% probability) or low-probability triplets (where the first and last elements are random; 37.5% probability). The 8-element sequences are repeated 10 times in one block. ASRT, Alternating Serial Reaction Time task.

In this task, participants tracked the appearance of a stimulus (dog's head), which continuously appeared in one of four circles arranged horizontally on the screen. Their objective was to respond as quickly and accurately as possible using the 's', 'f', 'j', 'l' keys on a QWERTZ keyboard. A block of the ASRT task included ten repetitions of an 8-element sequence, resulting in 80 stimuli within each block. The stimuli remained visible until a response was made. Following a correct answer, a 120 ms pause preceded the presentation of the next stimulus; if the answer was wrong, the stimulus stayed on display. At the conclusion of each block, participants received individual feedback on average accuracy and response time and were allowed to take a brief rest. The structure of the ASRT task is illustrated in Fig. 1.

The task began with one practice block comprising only random trials (80 stimuli). After this, participants progressed to complete the first session of the ASRT task, which contained 15 blocks. For analytical purposes, these blocks were divided into three bins, each containing five blocks. Following the first session, participants took a 10-minute break before continuing to the second session, which included one additional bin, thereby bringing the total to four bins across both sessions. The source code for these tasks is openly accessible via the link provided in the Data Availability Statement.

#### 2.2.2 Work addiction

We assessed work addiction risk using the Hungarian version of the Work Addiction Risk Test (WART; (Robinson, 1989; Urbán et al., 2019). This questionnaire is widely recognized for its reliability and validity and is among the most commonly used scales for measuring work addiction (Andersen et al., 2023, Clark et al., 2020, Robinson, 1999). Comprising 25 items rated on a four-point Likert scale (ranging from 'never true' to 'always true.'), it includes items such as “I feel guilty when I am not working on something”. We utilized the total score from the questionnaire, as it offers the most comprehensive overview of an individual's risk of work addiction. While the questionnaire was developed based on clinical observations, it should not be considered a diagnostic tool. In our sample, the scale demonstrated a good internal reliability (Cronbach's alpha = 0.87).

### 2.3 Procedure

We recruited participants who had previously participated in a previous research examining the relationship between work addiction and personality traits (Kun, Urban et al., 2020), selecting them based on their scores from the WART. In that study, participants indicated their willingness to engage in future research by providing contact information after completing the survey. However, WART scores were reassessed in the current study to account for potential variations in work addiction symptoms over time.

The two-hour-long face-to-face sessions started with an explanation of the research procedures and consent form signing, followed by questions about their socioeconomic status (SES), health and questionnaires relating to exclusion criteria, then neuropsychological tests. Out of these measures, for this study we used the WART score and the ASRT task data. Participants received gift vouchers valued at 25 EUR as compensation.

This study was conducted in accordance with ethical standards and received approval from the institutional Research Ethics Committee (registration number 2020/401). We adhered to the principles outlined in the Declaration of Helsinki throughout the study.

### 2.4 Statistical analysis

Statistical analyses were conducted using JASP (Version 0.18.3; JASP Team, 2023) and IBM SPSS Statistics (Version 28; IBM Corp., 2021). The plots were generated using Python (Version 3.10.12) using *pandas*, *io* and *matplotlib* packages (Hunter, 2007, McKinney, 2010). A priori power analysis was performed using G*Power version 3.1.9.4 (Faul et al., 2007) to estimate the required sample size.

First, we examined whether the LWA and HWA groups differed in gender, age, education, place of residence, current SES, and childhood SES. We used independent sample t-tests or Mann-Whitney tests for continuous variables, depending on normality assumptions, and Chi-squared tests for categorical variables. To account for the influence of potential confounding variables on WART scores, we controlled for age and current SES (see Supplementary S2).

We computed scores for the ASRT task analysis as follows: for each bin and each participant, we determined the median reaction time (RT) and mean accuracy (see further details on the preparation of data in Supplementary S1). We carried out two mixed-design analyses of variance (ANOVAs) to analyze the habit learning process. The dependent variables were accuracy and RT, respectively. The grouping variable was the two-level group, with levels representing the LWA and the HWA groups. One of the within-subject factors was triplet, which had two levels, high-probability and low-probability triplets; the other was bin, which had 4 levels. Where sphericity was impaired, we used the Greenhouse-Geisser correction. We also conducted Bayesian analyses and calculated the BF_incl_ values (for the interpretation of the bayes factors, see Supplementary S3).

To further investigate the effects of WART scores on habit learning, we performed two linear mixed models to explore the relationships between WART scores and learning outcomes (see Supplementary S5).

## 3 Results

The power analysis indicated that a total sample size of 90 was required to achieve a power of 0.90, demonstrating that our sample size of 104 is sufficient to attain statistical power (see further details in Supplementary Materials S4).

### 3.1 Is habit learning different in the HWA and LWA groups? Reaction time differences

**Fig. 2:**
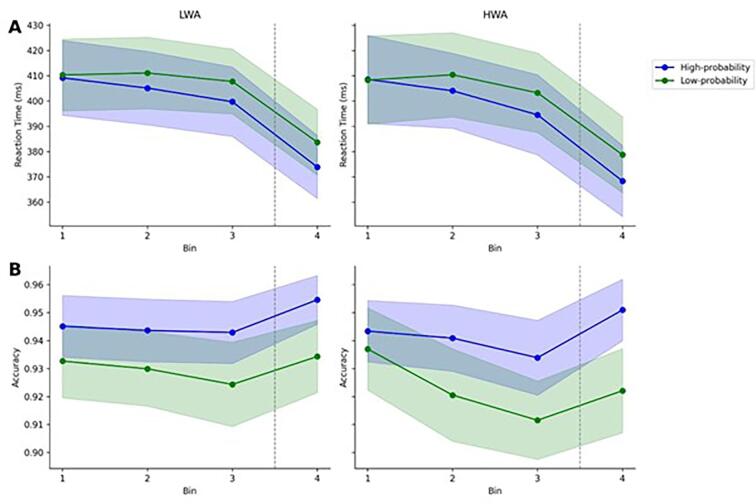
Reaction Times to High- and Low-Probability Triplets in the HWA and LWA Groups. *Note*. Figure A presents reaction time (RT), while Figure B shows accuracy data. In both figures, the left and right panels correspond to the Low risk for Work Addiction (LWA) and High risk for Work Addiction (HWA) groups. The green and blue lines represent RT and accuracies associated with high-probability and low-probability triplets. A more pronounced divergence between these two types of triplets serves as an indicator of enhanced statistical learning. Importantly, a gray dashed line delineates a 20-minute break in the procedure. To provide a measure of data dispersion, the shaded error bands in both figures represent the 95% confidence interval around the mean.

The mixed-design ANOVA showed no significant Triplet Type main effect when using current SES and age as covariates, indicating that by controlling for these variables, there was no habit learning in the groups in reaction time, as they reacted with similar reaction times to high- and low-probability triplets. The significant main effect of Bin showed that reaction time improved across time. However, the interactions between Triplet × Group, as well as Bin × Triplet × Group, did not reach statistical significance. This result suggests that there were no significant differences observed between the groups in the amount or in the patterns of learning (see Fig. 2.A). When considering covariates, the results showed that age had a significant interaction with triplet type, indicating that age differences had an effect on habit learning (for more details, see Table 1).

**Table 1:** Results of the Repeated Measures ANOVA Analysis on Reaction Time of the Alternating Serial Reaction Time Task.

Effects	F	df	p	η^2^p	BF_incl_
Triplet Type	0.31	1, 100	0.580	0.003	4.569 × 10^9^
Triplet Type * Current SES	0.39	1, 100	0.537	0.004	
Triplet Type * Age	5.10	1, 100	**0.026**	0.049	
Triplet Type * Group	0.09	1, 100	0.767	0.001	0.022
Bin	2.89	2.65, 265.32	**0.042**	0.028	5.806 × 10^60^
Bin * Current SES	0.70	2.65, 265.32	0.536	0.007	
Bin * Age	2.02	2.65, 265.32	0.119	0.020	
Bin * Group	1.86	2.65, 265.32	0.144	0.018	0.030
Triplet Type * Bin	1.58	2.94, 293.49	0.195	0.016	39469.470
Triplet Type * Bin * Current SES	1.64	2.94, 293.49	0.182	0.016	
Triplet Type * Bin * Age	1.70	2.94, 293.49	0.169	0.017	
Triplet Type * Bin * Group	0.04	2.94, 293.49	0.989	0.000	0.017

*Note*. Statistical significance at *p* < 0.05 is indicated by boldfacing.

### 3.2 Is habit learning different in the HWA and LWA groups? Accuracy differences

The mixed-design ANOVA with age and current SES as covariates revealed a significant main effect for Triplet Type on accuracy, suggesting that participants, regardless of their groups, displayed higher accuracy when responding to high-probability triplets compared to low-probability triplets, confirming the presence of habit learning. There was a tendency-level Bin × Triplet interaction, as participants distinguished triplets with increasing accuracy as the task progressed. Additionally, there was a tendency-level interaction between Bin and Group, indicating that the groups had a different pattern in accuracy. Consistent with the reaction time results, the absence of significant interactions between Triplet × Group and Bin × Triplet × Group suggests that both the amount and the patterns of learning were similar in the groups (see Fig. 2.B). When considering covariates, the results showed that age and current SES did not have an effect on triplet type or Bin (see Table 2).

**Table 2:** Results of the Repeated Measures ANOVA Analysis on the Accuracy of the Alternating Serial Reaction Time Task.

Effects	F	df	*p*	η^2^p	BF_incl_
Triplet Type	6.53	1, 100	**0.012**	0.061	1.379*10^12
Triplet Type * Current SES	0.26	1, 100	0.608	0.003	
Triplet Type * Age	0.91	1, 100	0.343	0.009	
Triplet Type * Group	0.32	1, 100	0.575	0.003	0.148
Bin	1.79	2.77, 276.55	0.154	0.018	147.352
Bin * Current SES	0.49	2.77, 276.55	0.672	0.005	
Bin * Age	1.49	2.77, 276.55	0.221	0.015	
Bin * Group	2.49	2.77, 276.55	0.066	0.024	0.060
Triplet Type * Bin	2.21	2.93, 293.20	0.088	0.022	2.199
Triplet Type * Bin * Current SES	0.67	2.93, 293.20	0.566	0.007	
Triplet Type * Bin * Age	1.79	2.93, 293.20	0.150	0.018	
Triplet Type * Bin * Group	1.15	2.93, 293.20	0.329	0.011	0.071

*Note*. Statistical significance at *p* < 0.05 is indicated by boldfacing.

The means and standard deviations of the accuracy and reaction time scores can be found in Supplementary Materials S6, Table S4. Linear mixed models using accuracy and RT as outcome variables showed the same results as ANOVA, with no association found between WART scores and habit learning (see Supplementary S5). In sum, this confirmed that there was no significant relationship between work addiction and habit learning when analyzed using two different statistical methods.

## 4 Discussion

Work addiction is predominantly characterized by an obsessive preoccupation with work, accompanied by excessive overinvolvement in work-related activities, often resulting in the neglect of other life aspects (Atroszko et al., 2019, Schaufeli et al., 2008). This phenomenon is associated with negative consequences, such as burnout, depressive symptoms, work-family conflict, and health problems (Chang et al., 2022, Clark et al., 2016, Dutheil et al., 2020, Serrano-Fernández et al., 2021). We presumed that the compulsive patterns of this behavioral addiction might be associated with a more habit-oriented functioning characterized by more automatic and repetitive behaviors (Demetrovics et al., 2022). Based on this assumption, we hypothesized that the dominance of the habitual system could be connected to work addiction, enhancing more automatized, sequence-like patterns (Dezfouli & Balleine, 2012). We examined habit learning in work addiction by using a probabilistic sequence learning task that primarily assesses the acquisition and automation of environmental regularities (Conway, 2020, Horváth et al., 2022). To the best of our knowledge, this was the first study examining the relationship between work addiction and habit learning.

Contrary to our hypothesis, we observed no enhanced habit learning in high risk for work addiction. This result stands in contrast to findings in substance use disorders, where an augmented habit learning is often noted, albeit in a context significantly influenced by drug use (Furlong & Corbit, 2018). Notably, research on other behavioral addictions, such as gaming disorder, gambling disorder, and internet addiction, points to an overreliance on habitual systems (Wyckmans et al., 2019; B. Zhou et al., 2018; W. Zhou et al., 2021). Research on other behavioral addictions, such as gaming disorder, gambling disorder, and internet addiction, as well as OCD, indicates an overreliance on habitual systems, with enhanced habit learning often observed alongside higher compulsiveness (Gillan et al., 2011, Gillan et al., 2016, Wyckmans et al., 2019, Zhou et al., 2018, Zhou et al., 2021). Understanding how compulsive tendencies in other behavioral addictions and OCD differ from those in work addiction is essential. An important difference compared to most of the earlier studies is that many of them used different kinds of tasks: outcome devolution tasks or contingency degradation tasks (Doñamayor et al., 2022), instead of sequence learning tasks used in this study. Here, we followed the suggestion of a previous study (Doñamayor et al., 2022), claiming that sequence learning is a promising avenue for exploring habit learning in addiction. These sequence learning tasks usually assess automatic visuomotor skills (Éltető et al., 2022), which can reveal excessively rigid behavioral patterns characteristic of addiction. Consequently, the absence of observed differences in individuals at high risk for work addiction may indicate a diminished role of these automatic visuomotor skills in this specific addiction.

The probabilistic sequence learning task used in this study is a typical model-free learning task and an established measure of predictive processes (Éltető et al., 2022, Pesthy et al., 2023). Our results suggest that the predictive processes of individuals with work addiction are fully intact and highly efficient. This may explain why work addiction shows more efficient functioning and better adaptation to the environment compared to substance use addictions and other behavioral addictions. Therefore, it is not a coincidence that work addiction is more accepted by society (however, the reasons behind this phenomenon are complex and beyond the scope of this study). More focused and specific neurocognitive studies on predictive processing in work addictions are warranted.

Our finding prompts the question: What other cognitive or psychological mechanisms might underpin the enduring nature of these compulsive patterns in work addiction, compelling individuals to persist in this behavior even in the absence of enhanced habit learning (Schaufeli et al., 2008, Spence and Robbins, 1992, van Wijhe et al., 2011)? While our study revealed intact habit learning, it is crucial to underscore that, notwithstanding this, habitual functioning might significantly contribute to sustaining the addiction cycle in work addiction. Studies highlight a common trajectory in the addiction cycle, wherein goal-directed functioning, initially associated with impulsivity, predominates in the initial phase, gradually giving way to more compulsive, habitual functioning over time (Demetrovics et al., 2022, Everitt and Robbins, 2016, Furlong and Corbit, 2018). In alignment with prior research (Demetrovics et al., 2022), advanced stages of addiction severity in behavioral addictions also often exhibit a shift towards more habitual functioning. Although the relationship between work addiction and habit learning has not been explored in previous research, existing evidence indicates a stronger link between compulsiveness and impulsivity in more advanced stages of work addiction, potentially driven by a shift toward habitual processes (Demetrovics et al., 2022).

It is also possible this shift may result not only from enhanced habitual functions, but also from the alterations in goal-directed processes, which could allow the habitual system to dominate (Furlong & Corbit, 2018). Studies on behavioral addictions and obsessive–compulsive disorders have highlighted the dominance of habitual behavioral functioning. Several of these findings suggest that the primary issue lies in the underactivity of goal-directed processes, with habitual functioning remaining intact (Gillan et al., 2015, Wyckmans et al., 2019). Devaluation sensitivity, the ability to stop or adjust behavior when a stimulus is no longer rewarding, is primarily linked to dysfunction in goal-directed processes. However, it is not directly associated with model-free, habit learning mechanisms (Gillan et al., 2015). Individuals with impaired goal-directed processes are less likely to stop habitual behaviors, while those with intact goal-directed functioning are more sensitive to devaluation and can adapt their behavior, as observed in studies on addictions and OCD (Gillan et al., 2011; B. Zhou et al., 2018). Previous research has shown weaker executive functions in work addiction (Berta et al., 2023), with individuals at high risk demonstrating weaker inhibitory control and reduced complex working memory. Given the alterations of goal-directed functions, it is plausible that the intact habit learning observed in this study does not exclude the possibility of habitual system predominance in work addiction. It is essential to stress that our study did not explore the interplay between goal-directed and habitual systems. Therefore, these hypotheses remain speculative, highlighting the need for future research to explore them further and understand the role of habit learning in work addiction.

Acknowledging the limitations of this study is important for a more comprehensive interpretation of our findings. An important direction for future research is to explore the association between habit learning and work addiction by controlling for work engagement, as work engagement plays a crucial role in distinguishing individuals with clinically significant work addiction from highly engaged “workaholics” who maintain effective functioning in daily life (Loscalzo & Giannini, 2017). Since our study did not assess work engagement, this limitation may have influenced our findings of intact habit learning. Additionally, future research should examine whether habitual processes are elicited by neutral stimuli or are specific to addiction-related contexts. Behavioral addictions sometimes involve cognitive processes that manifest only in relation to addiction-related actions (Antons and Brand, 2018, Yao et al., 2015). In the case of work addiction, the variability of occupations makes it challenging to define a standardized work-related stimulus. However, studying these processes in high-risk individuals within their workplace environments could provide valuable insights. Future studies should also investigate the interplay between goal-directed and habitual systems within individuals with work addiction, providing valuable insights into underlying mechanisms through comparative analysis of their balance within the same study design. Furthermore, we used a random convenience sampling method, limiting the generalizability of our findings to a broader population. The assessment of work addiction risk was exclusively conducted through the WART questionnaire due to the absence of a diagnostic tool for this condition, thus constraining the scope of our conclusions.

## 5 Conclusion

Our results suggest that the compulsive patterns observed in work addiction may not be directly linked to habitual processes. From a habit learning perspective, work addiction appears distinct from substance and behavioral addictions. While sharing some cognitive similarities, such as the underperformance of goal-directed functions (Berta et al., 2023), work addiction seems to maintain intact habit learning. While this distinction might contribute to the overall better functioning of individuals with work addiction in everyday life, it is crucial to emphasize that they still experience significant negative consequences. Despite the unexpected findings, habitual functioning could still play a significant role in perpetuating the addiction cycle. A shift in the balance between the two systems can occur solely due to the underfunctioning of goal-directed processes, a characteristic frequently observed in work addiction. Future research needs to disentangle the intricate interplay between cognitive processes, refining our understanding of patterns contributing to work addiction's development and persistence. Additionally, future studies can clarify the role of work engagement in the link between habit learning and work addiction using validated measures and statistical controls, helping to distinguish maladaptive work addiction from high but non-pathological work involvement.

## CRediT authorship contribution statement

**Zsuzsanna Viktória Pesthy:** Writing – original draft, Visualization, Project administration, Methodology, Formal analysis, Data curation. **Krisztina Berta:** Writing – original draft, Visualization, Project administration, Methodology, Formal analysis, Data curation. **Teodóra Vékony:** Writing – review & editing, Supervision, Software, Methodology, Formal analysis. **Dezső Németh:** Writing – review & editing, Supervision, Resources, Methodology, Funding acquisition, Formal analysis, Conceptualization. **Bernadette Kun:** Writing – review & editing, Supervision, Resources, Project administration, Methodology, Investigation, Funding acquisition, Data curation, Conceptualization.

## Funding

This research was supported by the ANR grant awarded within the framework of the Inserm CPJ (Grant number: ANR-22-CPJ1–0042–01ANR); the National Brain Research Program by Hungarian Academy of Sciences (project NAP2022-I-1/2022); and the Hungarian National Research, Development and Innovation Office (Grant numbers: K128016 and FK134807) and by the ÚNKP-23–2 and ÚNKP-23–3 New National Excellence Program and the EKÖP-24 University Excellence Scholarship Program of the Ministry for Culture and Innovation from the source of the National Research, Development and Innovation Fund. Bernadette Kun was supported by the János Bolyai Research Scholarship of the Hungarian Academy of Sciences.

## Declaration of competing interest

The authors declare that they have no known competing financial interests or personal relationships that could have appeared to influence the work reported in this paper.

## Data Availability

All data are available on the following link: https://osf.io/ex5mp/.
