# Supplementary material for: Intact habit learning in work addiction: Evidence from a probabilistic sequence learning task
Source: Addict Behav Rep. 2025 Feb 10;21:100589. doi: 10.1016/j.abrep.2025.100589 (PMC11874818; doi:10.1016/j.abrep.2025.100589)
Supplement: Supplementary Data 1 [file mmc1.docx]

**Supplementary Materials**

**S1. The Alternating Structure of the Alternating Serial Reaction Time (ASRT) Task**

Triplets such as 3_2, 2_1, 1_4, and 4_3 (where "_" denotes the triplet's middle element) are expected to appear with high frequency (termed high-probability triplets). This is because the triplet's last element might be a part of the established pattern or could alternatively be selected from the random elements, similar to the triplet's first element. Conversely, triplets like 1_3 and 4_1 are predicted to have a lower occurrence (classified as low-probability triplets) since their first and last elements are exclusively random. As a result, the predictability of the concluding elements in high-probability triplets surpasses that in low-probability triplets due to the former appearing five times more often than the latter. In terms of occurrence rates, high-probability triplets are seen with a 4% probability at each keystroke and 62.5% across the entire block, while low-probability triplets manifest at a rate of 0.8% per keystroke and aggregate to 37.5% (see Fig. 1.). The disparity in occurrence rates between these triplet types forms the basis for measuring the level of learning.

 In our analysis, we specifically excluded pattern types known as 'trills'—sequential repetitions of alternating numbers (e.g., 1-2-1), akin to the musical trill where a rapid alternation between two adjacent notes occurs. Additionally, we removed instances of direct repetitions (e.g., 2-2-2), as both trills and direct repetitions might not accurately reflect the cognitive process we aimed to measure. This exclusion was based on the consideration that participants could have preexisting propensities towards these patterns, potentially skewing the data and affecting the integrity of our findings. Additionally, incorrect answers were excluded from the RT analysis.

**S2. Comparing Groups Along Sociodemographic Data**

First, we assessed the normality of continuous variables in the HWA and LWA groups, including age, current socioeconomic status (SES), and childhood SES, using the Shapiro-Wilk test. Depending on the results, our analysis strategy involved independent sample t test for variables conforming the normality distribution assumptions and Mann-Whitney test for those diverging from normality. The Shapiro-Wilk test revealed deviations from normality for current SES (*p* > .016) and childhood SES (*p* > .110); therefore, we employed Mann-Whitney tests. For age, normality was not violated (*p* > .098), so we performed an independent sample t-test. A significant difference in current SES and age was found between the groups (see Table S1).

We examined differences between the groups across categories for categorical variables including gender, education, and place of residence with Chi square tests. We did not find significant differences in any of the categories (see Table S1).

**Table S1**

*Comparison of HWA and LWA groups in Age, Childhood SES, Current SES, Gender, Educational Level and Place of Residence*

| Variable | Statistics   t/ U/χ2 | *df* | *p* |
| --- | --- | --- | --- |
| Age | 2.302 | 102 | **.023** |
| Childhood SES | 1364 | - | .568 |
| Current SES | 1628 | - | **.018** |
| Gender | 3.3 | 1 | .069 |
| Education | 0.968 | 3 | .809 |
| Place of residence | 4.627 | 3 | .201 |

*Note.* Statistical significance at *p* < 0.05 is indicated by boldfacing. SES, socioeconomic status. LWA, Low Risk of Work Addiction group; HWA, High Risk of Work Addiction group.

**S3. Interpretation of the Bayes Factors**

The interpretation of Bayes factors was based on Jeffreys' (1998) categories of evidence. According to these categories, Bayes factors between 1 and 3 indicate anecdotal evidence, values between 3 and 10 suggest significant evidence, and values greater than 10 indicate strong evidence in favor of the alternative hypothesis (Wagenmakers et al., 2011).

**S4. Power analysis**

Since there are similar studies using the ASRT task reporting sample sizes, we used the effect size from Janacsek et al. (2018), which employed this task with individuals experiencing major depressive episodes, given that depressive episodes are often comorbid with work addiction (Serrano-Fernández et al., 2021). We used the effect size of the three-way interaction from this study (η²p = 0.052). The results indicated that a total sample size of 90 was required to achieve a power of 0.90. This demonstrates that our sample size of 104 is sufficient to attain this statistical power.

**S5. Linear Mixed Models with Covariates**

To investigate the effect of WART scores on habit learning, we performed two linear mixed models with the median RT and mean accuracy as outcome variables, respectively. The models included Bin (1-4), Triplet Type (high- vs. low-probability triplets), WART score and their interactions as fixed effects. The model incorporated participants as random intercepts and included by-participant slopes for the Bin factor, allowing for variability in general speed-up over time. It is important to note that the main effect of Triplet Type and its interactions suggest differences in habit learning. In contrast, the presence of effects and interactions without this suggests a difference in visuomotor learning, which means only the improvements in speed or accuracy on the task.

**S5.1. Reaction Time**

The model on reaction times revealed a significant main effect of Triplet Type, indicating quicker reaction times for high-probability triplets compared to low-probability triplets, underscoring the occurrence of habit learning. Additionally, the model reported a main effect of Bin, indicating decreasing reaction time throughout the task. The model also revealed a significant interaction between Triplet Type and Bin, suggesting that the magnitude of habit learning fluctuated over time. No significant associations were found with the main effect of WART score or other interactions, suggesting that WART scores did not significantly influence reaction times in the ASRT task (see Supplementary Table S2).

**Table S2.**

*The Results of the Linear Mixed Model, Predicting Median Reaction Time of the Alternating Serial Reaction Time Task*

| Effect | std. b | std. 95% CI | *df* | *F* | *p* |
| --- | --- | --- | --- | --- | --- |
| Triplet Type | –0.06 | [–0.07, –0.05] | 1, 408.00 | 97.48 | **<.001***** |
| WART score | –0.10 | [–0.28, 0.08] | 1, 102.01 | 1.11 | .295 |
| Bin 1 | 0.19 | [0.15, 0.22] | 1, 102.00 | 114.46 | **<.001** |
| Bin 2 | 0.17 | [0.14, 0.20] | 1, 102.00 | 114.46 | **<.001** |
| Bin 3 | 0.06 | [0.02, 0.09] | 1, 102.00 | 114.46 | **<.001** |
| Triplet Type × WART score | –0.00 | [–0.02, 0.01] | 1, 408.00 | 0.62 | .431 |
| Triplet Type × Bin 1 | 0.05 | [0.03, 0.07] | 3, 408.00 | 10.12 | **<.001** |
| Triplet Type × Bin 2 | 0.00 | [–0.02, 0.02] | 3, 408.00 | 10.12 | **<.001** |
| Triplet Type × Bin 3 | –0.02 | [–0.04, 0.00] | 3, 408.00 | 10.12 | **<.001** |
| WART score × Bin 1 | 0.01 | [–0.03, 0.05] | 3, 102.00 | 0.30 | .828 |
| WART score × Bin 2 | 0.01 | [–0.02, 0.04] | 3, 102.00 | 0.30 | .828 |
| WART score × Bin 3 | –0.01 | [–0.04, 0.02] | 3, 102.00 | 0.30 | .828 |
| Triplet Type × WART score × Bin 1 | 0.00 | [–0.02, 0.02] | 3, 408.00 | 0.20 | .895 |
| Triplet Type × WART score × Bin 2 | 0.00 | [–0.02, 0.03] | 3, 408.00 | 0.20 | .895 |
| Triplet Type × WART score × Bin 3 | –0.00 | [–0.02, 0.02] | 3, 408.00 | 0.20 | .895 |

*Note.* WART, Work Addiction Risk Test. Bins are compared to the fourth bin, which is implied as a baseline or reference category in the comparisons of Bins 1, 2, and 3.

**S5.2. Accuracy**

The model on mean accuracy revealed a significant main effect of Triplet Type. Participants exhibited higher accuracy to high-probability than to low-probability triplets, indicating the presence of habit learning among the participants in accuracy measures as well. Furthermore, the model revealed a significant main effect of Bin and interaction between Triplet Type and Bin, implying that the accuracy and the extent of habit learning changed over time. The WART score did not have a significant main effect and was not involved in any significant interactions, indicating that it did not have a connection with the reaction times in the ASRT task (For more details, see Table S3).

**Table S3.**

*The Results of the Linear Mixed Model, Predicting Mean Accuracy of the ASRT Task*

| Effect | std. b | std. 95% CI | *df* | *F* | *p* |
| --- | --- | --- | --- | --- | --- |
| Triplet Type | 0.19 | [0.15, 0.22] | 1, 510.01 | 127.79 | **<.001** |
| WART score | –0.08 | [–0.24, 0.08] | 1, 102.00 | 0.87 | .352 |
| Bin 1 | 0.07 | [–0.01, 0.14] | 3, 130.83 | 01.júl | **<.001** |
| Bin 2 | –0.04 | [–0.11, 0.03] | 3, 130.83 | 01.júl | **<.001** |
| Bin 3 | –0.14 | [–0.21, –0.07] | 3, 130.83 | 01.júl | **<.001** |
| Triplet Type × WART score | 0.03 | [–0.01, 0.06] | 1, 510.01 | 2.47 | .117 |
| Triplet Type × Bin 1 | –0.08 | [–0.14, –0.02] | 3, 510.01 | 3.49 | **.016** |
| Triplet Type × Bin 2 | –0.01 | [–0.06, 0.05] | 3, 510.01 | 3.49 | **.016** |
| Triplet Type × Bin 3 | 0.02 | [–0.03, 0.08] | 3, 510.01 | 3.49 | **.016** |
| WART score × Bin 1 | 0.00 | [–0.07, 0.08] | 3, 130.83 | 0.34 | .798 |
| WART score × Bin 2 | 0.01 | [–0.06, 0.08] | 3, 130.83 | 0.34 | .798 |
| WART score × Bin 3 | –0.03 | [–0.10, 0.04] | 3, 130.83 | 0.34 | .798 |
| Triplet Type × WART score × Bin 1 | –0.03 | [–0.08, 0.03] | 3, 510.01 | 0.60 | .615 |
| Triplet Type × WART score × Bin 2 | –0.01 | [–0.07, 0.04] | 3, 510.01 | 0.60 | .615 |
| Triplet Type × WART score × Bin 3 | 0.01 | [–0.05, 0.07] | 3, 510.01 | 0.60 | .615 |

*Note.* WART, Work Addiction Risk Test. Bins are compared to the fourth bin, which is implied as a baseline or reference category in the comparisons of Bins 1, 2, and 3.

**S6. Means and standard deviations of the accuracy and reaction time scores in the ASRT task.**

**Table S4**

*Performance of the HWA and LWA Inaccuracy and Reaction Time of the ASRT Task*

| Measure | LWA | | HWA | |
| --- | --- | --- | --- | --- |
|  | Mean | SD | Mean | SD |
| H1 ACC | 0.947 | 0.043 | 0.943 | 0.034 |
| H2 ACC | 0.946 | 0.044 | 0.941 | 0.037 |
| H3 ACC | 0.945 | 0.043 | 0.934 | 0.042 |
| H4 ACC | 0.957 | 0.034 | 0.951 | 0.034 |
| L1 ACC | 0.935 | 0.051 | 0.937 | 0.046 |
| L2 ACC | 0.932 | 0.052 | 0.921 | 0.052 |
| L3 ACC | 0.927 | 0.059 | 0.912 | 0.044 |
| L4 ACC | 0.937 | 0.050 | 0.922 | 0.047 |
| H1 RT | 411.27 | 57.55 | 408.54 | 54.49 |
| H2 RT | 408.01 | 57.57 | 404.04 | 46.42 |
| H3 RT | 402.45 | 54.30 | 394.53 | 49.58 |
| H4 RT | 376.54 | 49.53 | 368.35 | 44.02 |
| L1 RT | 412.54 | 55.39 | 408.30 | 54.52 |
| L2 RT | 414.23 | 56.45 | 410.37 | 51.99 |
| L3 RT | 410.54 | 50.95 | 403.24 | 49.16 |
| L4 RT | 386.52 | 51.68 | 378.74 | 47.07 |

*Note*. ASRT, Alternating Serial Reaction Time task; ACC, accuracy; H, high-probability triplets; L, low-probability triplets, the number after the latter indicates the number of the bin; LWA, Low Risk of Work Addiction group; HWA, High Risk of Work Addiction group; RT, the average reaction time for the triplets (in ms).

**References**

Janacsek, K., Borbély-Ipkovich, E., Nemeth, D., & Gonda, X. (2018). How can the depressed mind extract and remember predictive relationships of the environment? Evidence from implicit probabilistic sequence learning. *Progress in Neuro-Psychopharmacology and Biological Psychiatry*, *81*, 17–24. <https://doi.org/10.1016/j.pnpbp.2017.09.021>

Jeffreys, H. (1998). *The theory of probability*. OuP Oxford.

Kumle, L., Võ, M. L.-H., & Draschkow, D. (2021). Estimating power in (generalized) linear mixed models: An open introduction and tutorial in R. *Behavior Research Methods, 53*(6), 2528–2543. <https://doi.org/10.3758/s13428-021-01546-0>

Lafit, G., Adolf, J.K., Dejonckheere, E., Myin-Germeys, I., Viechtbauer, W., Ceulemans, E. (2021). Selection of the Number of Participants in Intensive Longitudinal Studies: A User-Friendly Shiny App and Tutorial for Performing Power Analysis in Multilevel Regression Models That Account for Temporal Dependencies. *Advances in Methods and Practices in Psychological Science,* 4(1). <https://doi.org/10.1177/2515245920978738>

Serrano-Fernández, M.-J., Boada-Grau, J., Boada-Cuerva, M., & Vigil-Colet, A. (2021). Work addiction as a predictor of anxiety and depression. *Work*, *68*(3), 779–788. <https://doi.org/10.3233/WOR-203411>

Wagenmakers, E.-J., Wetzels, R., Borsboom, D., & Van Der Maas, H. L. J. (2011). Why psychologists must change the way they analyze their data: The case of psi: Comment on Bem (2011). *Journal of Personality and Social Psychology*, *100*(3), 426–432. https://doi.org/10.1037/a0022790
